# Association of digestive symptoms with severity and mortality of COVID-19: A protocol for systematic review and meta-analysis

**DOI:** 10.1097/MD.0000000000022736

**Published:** 2020-10-23

**Authors:** Yufang Zhang, Peifen Ma, Xiu Zhang, Zhuoxi Pei, Haixia Wang, Xinman Dou

**Affiliations:** a Department of Nursing; b The Second Ward of Orthopedics Department; c The First Ward of Urology Department; d The First Ward of Cardiovascular Medicine Department, Lanzhou University Second Hospital, Lanzhou, China.

**Keywords:** COVID-19, gastrointestinal symptoms, meta-analysis, mortality, SARS-CoV-2, severity

## Abstract

**Background:**

Gastrointestinal manifestations are common in patients with COVID-19, but the association between specific digestive symptoms and COVID-19 prognosis remains unclear. This study aims to assess whether digestive symptoms are associated with COVID-19 severity and mortality.

**Methods:**

We will search PubMed, Embase, Web of Science, and the Cochrane Central Register of Controlled Trials up to September, 2020, to identify studies that compared the prevalence of at least one specific digestive symptom between severe and non-severe COVID-19 patients or between non-survivors and survivors. Two independent reviewers will assess the risk of bias of the included cohort studies using the modified Newcastle-Ottawa Scale. Meta-analyses will be conducted to estimate the pooled prevalence of individual symptoms using the inverse variance method with the random-effects model. We will conduct subgroup analyses, sensitivity analyses, and meta-regression analyses to explore the sources of heterogeneity. The Grading of Recommendations Assessment, Development, and Evaluation (GRADE) approach will be used to assess the quality of the evidence.

**Results:**

The results of this study will be published in a peer-reviewed journal.

**Conclusion:**

Our meta-analysis will comprehensively evaluate the association between different digestive symptoms and the severity and mortality of patients infected with COVID-19. This study will provide evidence to help determine whether special protective measures and treatment options are needed for patients with digestive system comorbidities during the COVID-19 pandemic.

**INPLASY registration number:**

INPLASY202090055.

## 1 Introduction

Coronavirus disease 2019 (COVID-19) caused by severe acute respiratory syndrome coronavirus 2 (SARS-CoV-2) infection has become a pandemic.^[1]^ The number of infection cases continues to rise, posing huge challenges to global public health. As of August 31, 2020, a total of 24,854,140 laboratory-confirmed cases and 838,924 deaths were reported globally, with a mortality rate of 3.4%.^[2]^

It is well established that common symptoms of COVID-19 patients are respiratory symptoms, such as dyspnea and cough.^[3–5]^ Apart from respiratory symptoms, patients with COVID-19 often have gastrointestinal manifestations.^[6]^ Several studies have shown that gastrointestinal symptoms in patients with COVID-19 are associated with poor progression and prognosis.^[3,7,8]^ Previous meta-analyses also explored the association between gastrointestinal manifestations and the severity of COVID-19 infection.^[9–11]^ But some results in these meta-analyses were inconsistent and the association between specific digestive symptoms, such as abdominal pain and constipation, and COVID-19 prognosis remains unclear. Furthermore, these meta-analyses drew conclusions based on limited sample sizes and almost all evidence from China. Therefore, it is necessary to conduct a comprehensive meta-analysis to generate more valid evidence to support clinical practice. This meta-analysis will assess whether digestive symptoms are associated with COVID-19 severity and mortality.

## 2 Methods

This systematic review will be performed in accordance with the Preferred Reporting Items for Systematic Reviews and Meta-Analyses (PRISMA) statement.^[12]^ The review protocol has been registered in the International Platform of Registered Systematic Review and Meta-Analysis Protocols (INPLASY, INPLASY202090055, doi: 10.37766/inplasy2020.9.0055).

### 2.1 Search strategy

We will search PubMed, Embase, Web of Science, and the Cochrane Central Register of Controlled Trials (CENTRAL) to identify clinical studies using search terms “coronavirus disease-19”, “coronavirus disease 2019”, “COVID-19”, “2019-nCoV”, “novel corona virus”, “novel coronavirus”, “new coronavirus”, “nCoV-2019”, “novel coronavirus pneumonia”, “2019 novel coronavirus”, “severe acute respiratory syndrome coronavirus 2”, “SARS-CoV-2”, “clinical characteristic”, “clinical feature”, “risk factor”, “prognosis”, “nausea”, “vomiting”, “diarrhea”, “digestive symptom”, and “gastrointestinal symptom”. The searches will be conducted in September 2020. The detailed search strategy of PubMed is presented in Table 1. We will also manually search reference lists of eligible studies and relevant systematic reviews to identify additional potentially eligible studies.

**Table 1:**
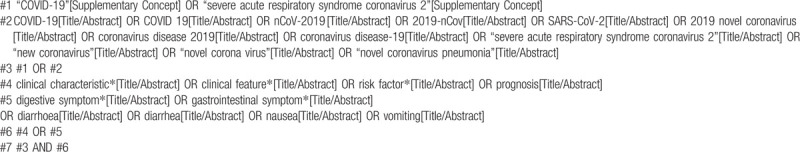
Search strategy of PubMed.

### 2.2 Inclusion and exclusion criteria

Our meta-analysis will include clinical studies that met the following criteria:

1. patients should be diagnosed with COVID-19 by a laboratory test;
2. provided the prevalence of at least 1 specific digestive symptom in infected patients;
3. compared patients with the severe and non-severe disease or between non-survivors and survivors;
4. written in English or Chinese;
5. with a sample size of larger than 20 patients.

We will exclude studies with following characteristics:

1. did not provide the prevalence of digestive symptoms;
2. only provided the overall prevalence of digestive symptoms without a detailed digestive symptom;
3. without comparisons (e.g., non-survivors versus survivors);
4. involved suspected cases;
5. reviews, abstracts, and editorials.

### 2.3 Study selection process

We will adopt EndNote X8 (Thomson Reuters (Scientific) LLC Philadelphia, PA, US) software to manage the retrieved records and detect duplicates. Two reviewers will independently (YFZ and PFM) screen the titles and abstracts of records. Full texts of potentially eligible studies will be evaluated to determine the eligibility of each study according to the inclusion criteria. Regarding multiple studies with overlapping data, we will include the study with larger sample size. Disagreements will be resolved through discussions with a third reviewer (XMD).

### 2.4 Data extraction

We will develop a standardized data extraction form using Microsoft Excel 2016 (Microsoft Corp, Redmond, WA, www.microsoft.com) through discussions with the review team and will revise the content after piloting on a random of 5 studies. We will extract the following information from included studies: first author, country of the first author, journal name, year of publication, publication language, study setting, recruitment time frame; age and sex of patients, sample size; prevalence of digestive symptoms, including diarrhea, nausea, vomiting, anorexia, abdominal pain, bloating, and constipation; number of severe cases, non-severe cases, non-survivors, and survivors. Disease severity will be defined as patients with acute respiratory distress syndrome (ARDS), requiring vital life support, needing mechanical ventilation, or needing intensive care unit admission (ICU) care.^[13,14]^

### 2.5 Risk of bias assessment

The Newcastle-Ottawa quality assessment scale (NOS) will be used to assess the quality of included studies.^[15]^ We will consider studies with more than 7 stars as high quality, 5 to 7 stars as moderate quality, and lower than 5 stars as low quality. Two independent reviewers (YFZ, PFM, XZ, or ZXP) will conduct data extraction and quality assessment and a third reviewer will check the data (HXW). Discrepancies will be resolved by consensus or by the discussion with a third reviewer (XMD).

### 2.6 Statistical analysis

We will perform meta-analyses to calculate the odds ratio (OR) and 95% confidence interval (CI) to estimate the association between digestive symptoms and COVID-19 severity and mortality using the inverse variance method. Owing to heterogeneity within and between studies, meta-analyses will be conducted using the random-effects model. The Cochran's Q test and *I*^2^ statistic will be used to assess the statistical heterogeneity, *I*^2^ values of <25%, 26% to 50%, and >50% will be considered as low, moderate, and high degrees of heterogeneity, respectively.^[16]^ If substantial heterogeneity is identified among studies, we will conduct subgroup analyses, sensitivity analyses, and meta-regression analyses to explore the sources of heterogeneity. All statistical analyses will be performed with Stata (13.0; Stata Corporation, College Station, Texas, USA Stata) and the statistical level of significance is set at *P* < .05.

### 2.7 Subgroup analysis, sensitivity analysis, and meta-regression analysis

Sensitivity analyses will be conducted by excluding studies published in Chinese or studies with high risk of bias to assess the stability of results. Subgroup analyses will be conducted for outcomes between different countries to explore the potential sources of heterogeneity. We will further perform univariate meta-regression analyses to assess if either the outcomes or the heterogeneity is associated with the sample size of studies included.

### 2.8 Publication bias

We will evaluate the publication bias using the funnel plot and Eggers test for outcomes with studies more than 9.

### 2.9 Certainty of evidence

We will assess the certainty of evidence for each meta-analysis using the Grading of Recommendations Assessment, Development and Evaluation (GRADE) framework, which includes 5 criteria: risk of bias, inconsistency, imprecision, indirectness, and publication bias.^[17–19]^ We will rate the quality of evidence as high, moderate, low, or very low and will present the results in the Summary of Findings table.

## 3 Results

### 3.1 Screening results

**Figure 1:**
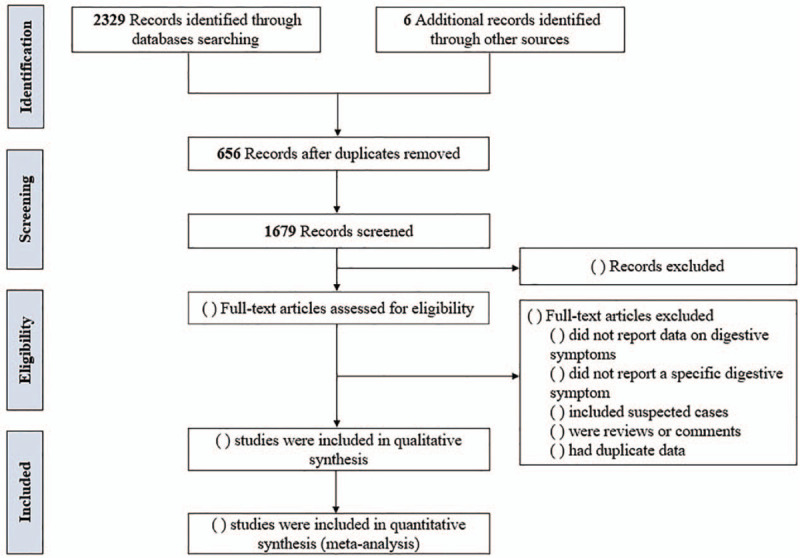
PRISMA flow chart for the included studies.

We conducted preliminary searches and 2335 records were identified. After removing duplicates, 1679 records remained. Then, we will conduct titles, abstracts, and full-text screening. The PRISMA flow chart for the screening process of the included studies is presented in Figure 1. In this protocol, we included 11 studies to conduct a pilot analysis.^[7,20–22,23–29]^

### 3.2 General characteristics

In the pilot analysis, we included 11 studies. The main characteristics of the included studies are shown in Table 2. All studies published in 2020, most studies were from China except for 1 from the USA.^[23]^ The sample size of individual studies ranged from 34 to 663 (total 2445; 1235 males).

**Table 2:**
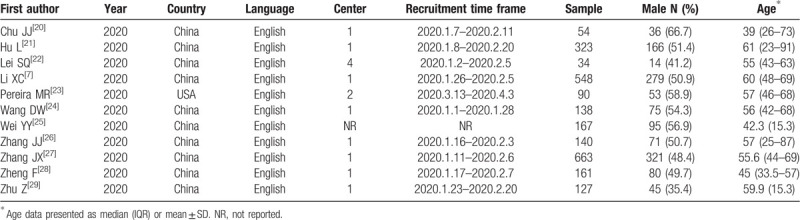
Characteristics of included studies.

### 3.3 Association between digestive symptoms and the severity of COVID-19

**Figure 2:**
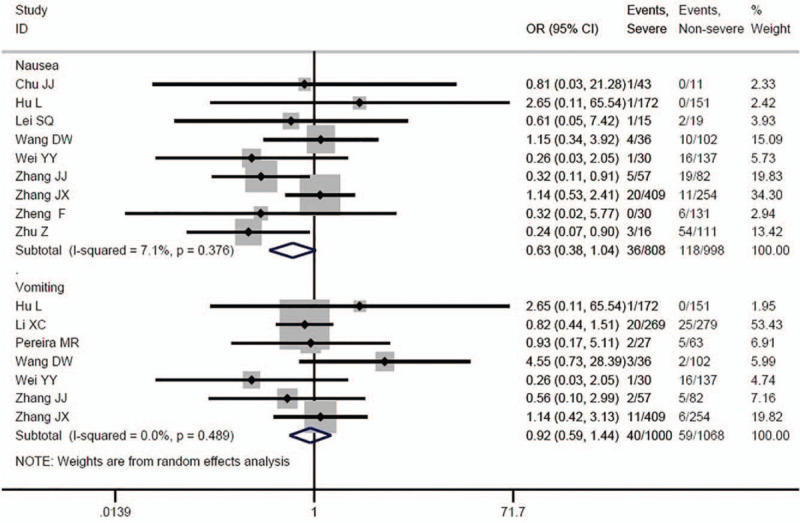
Association between nausea, vomiting and COVID-19 severity.

Some of the results are shown in Figure 2. No significant differences were found in the prevalence of nausea (OR = 0.63, 95%CI: 0.38–1.04, *P* = .071; *I*^2^ = 7.1%) and vomiting (OR = 0.92, 95%CI: 0.59–1.44, *P* = .717; *I*^2^ = 0.0%) between severe and non-severe patients.

## 4 Discussion

The previous evidence showed that some patients with COVID-19 initially exhibit gastrointestinal symptoms without fever or respiratory symptoms.^[30]^ The diagnosis of COVID-19 in these patients is usually delayed, thereby delaying the treatment time.^[9]^ Similarly, having a reliable estimate of the association between digestive symptoms and COVID-19 severity or mortality is also crucial to ensure specific successful global preventive and treatment strategies for COVID-19 patients.^[31]^ Our meta-analysis will comprehensively evaluate the association between different digestive symptoms and the severity and mortality of patients infected with COVID-19. Systematic reviews can provide information for health care management and policy making by providing research-based answers to important questions about the health system.^[32]^ We believe the results of this study will provide evidence to help determine whether special protective measures and treatment options are needed for patients with digestive system comorbidities during the COVID-19 pandemic.

## Author contributions

YFZ, PFM, XZ, ZXP, HXW, and XMD planned and designed the research. YFZ, PFM, XZ, ZXP, and HXW tested the feasibility of the study. PFM and XMD provided methodological advice, polished and revised the manuscript. YFZ and XMD wrote the manuscript. All authors approved the final version of the manuscript.

**Conceptualization:** Yufang Zhang, Peifen Ma, Xiu Zhang, Xinman Dou.

**Investigation:** Yufang Zhang, Peifen Ma, Xiu Zhang, Zhuoxi Pei, Haixia Wang, Xinman Dou.

**Methodology:** Peifen Ma, Zhuoxi Pei, Haixia Wang.

**Project administration:** Xinman Dou.

**Resources:** Peifen Ma, Xiu Zhang, Haixia Wang.

**Software:** Yufang Zhang.

**Supervision:** Xinman Dou.

**Validation:** Zhuoxi Pei, Xinman Dou.

**Visualization:** Yufang Zhang, Peifen Ma, Xiu Zhang.

**Writing – original draft:** Yufang Zhang, Xinman Dou.

**Writing – review & editing:** Yufang Zhang, Xinman Dou.
